# *Irx3* and *Irx5* - Novel Regulatory Factors of Postnatal Hypothalamic Neurogenesis

**DOI:** 10.3389/fnins.2021.763856

**Published:** 2021-11-02

**Authors:** Zhengchao Dou, Joe Eun Son, Chi-chung Hui

**Affiliations:** ^1^Program in Developmental & Stem Cell Biology, The Hospital for Sick Children, Toronto, ON, Canada; ^2^Department of Molecular Genetics, University of Toronto, Toronto, ON, Canada

**Keywords:** FTO (Fat Mass and Obesity-Associated) Gene, obesity, metabolic regulation, neural stem cell (NSC), tanycyte, neurogenesis, *IRX3* gene, *IRX5* gene

## Abstract

The hypothalamus is a brain region that exhibits highly conserved anatomy across vertebrate species and functions as a central regulatory hub for many physiological processes such as energy homeostasis and circadian rhythm. Neurons in the arcuate nucleus of the hypothalamus are largely responsible for sensing of peripheral signals such as leptin and insulin, and are critical for the regulation of food intake and energy expenditure. While these neurons are mainly born during embryogenesis, accumulating evidence have demonstrated that neurogenesis also occurs in postnatal-adult mouse hypothalamus, particularly in the first two postnatal weeks. This second wave of active neurogenesis contributes to the remodeling of hypothalamic neuronal populations and regulation of energy homeostasis including hypothalamic leptin sensing. Radial glia cell types, such as tanycytes, are known to act as neuronal progenitors in the postnatal mouse hypothalamus. Our recent study unveiled a previously unreported radial glia-like neural stem cell (RGL-NSC) population that actively contributes to neurogenesis in the postnatal mouse hypothalamus. We also identified *Irx3* and *Irx5*, which encode *Iroquois* homeodomain-containing transcription factors, as genetic determinants regulating the neurogenic property of these RGL-NSCs. These findings are significant as *IRX3* and *IRX5* have been implicated in *FTO*-associated obesity in humans, illustrating the importance of postnatal hypothalamic neurogenesis in energy homeostasis and obesity. In this review, we summarize current knowledge regarding postnatal-adult hypothalamic neurogenesis and highlight recent findings on the radial glia-like cells that contribute to the remodeling of postnatal mouse hypothalamus. We will discuss characteristics of the RGL-NSCs and potential actions of *Irx3* and *Irx5* in the regulation of neural stem cells in the postnatal-adult mouse brain. Understanding the behavior and regulation of neural stem cells in the postnatal-adult hypothalamus will provide novel mechanistic insights in the control of hypothalamic remodeling and energy homeostasis.

## Introduction

Obesity is a global health threat with increased risk for various chronic conditions including diabetes and cardiovascular diseases (Williams et al., 2015; Gonzalez-Muniesa et al., 2017). Although lifestyle changes have driven its prevalence to epidemic proportions, genetic factors play an important role in influencing which individuals within a population are more likely to develop obesity in response to a particular environment (Elks et al., 2012; van der Klaauw and Farooqi, 2015; Gonzalez-Muniesa et al., 2017). In the last few decades, tremendous efforts have been made to identify genetic factors involved in obesity. Among genetic variations in the human genome, single-nucleotide polymorphisms (SNPs) in the first intron of the Fat mass and obesity-associated gene (*FTO*) were identified to be most highly associated with obesity risk (Dina et al., 2007; Frayling et al., 2007; Cecil et al., 2008; Speakman et al., 2008; Tanofsky-Kraff et al., 2009). Recent studies further demonstrated that two homeobox genes, *IRX3* and *IRX5*, in the vicinity of *FTO* directly mediate the effects of obesity-risk variants of *FTO* on body mass and composition regulation (Smemo et al., 2014; Claussnitzer et al., 2015; Laber et al., 2021; Sobreira et al., 2021).

The hypothalamus is a central regulatory hub for many physiological processes including energy homeostasis. Especially, the hypothalamic arcuate-median eminence (ARC-ME) is involved in the sensing of various signals such as the satiety hormone leptin (Dietrich and Horvath, 2013; Timper and Bruning, 2017; Friedman, 2019; Myers et al., 2021). It contains major classes of leptin-sensing neurons including those expressing orexigenic agouti-related peptide (AgRP) and anorexigenic pro-opiomelanocortin (POMC), radial glia-like cells (RGLs) (tanycytes, ependymocytes and neural stem cells) as well as NG2^+^ oligodendrocyte precursor cells (OPCs) (Dietrich and Horvath, 2013; Campbell et al., 2017; Rizzoti and Lovell-Badge, 2017; Timper and Bruning, 2017). *Irx3* and *Irx5* are expressed in multiple cell types of the ARC-ME, predominantly in a newly identified radial glia-like neural stem cell (RGL-NSC) population in the postnatal mouse hypothalamus (Smemo et al., 2014; Campbell et al., 2017; Son et al., 2021a). They are implicated in the regulation of energy homeostasis, particularly feeding regulation, and postnatal hypothalamic neurogenesis (Son et al., 2021a). The role of IRX3 and IRX5 in the regulation of energy homeostasis and development has been previously discussed in other reviews (Cavodeassi et al., 2001; Gomez-Skarmeta and Modolell, 2002; Kim et al., 2012; Tung et al., 2014; Herman and Rosen, 2015; de Araujo and Velloso, 2020). In this review, we aim to provide an update integrating our knowledge of their new functions in hypothalamic neurogenesis and discuss the intricacies and challenges in understanding their molecular actions.

## *IRX3* and *IRX5* Homeobox Genes are Effectors of *FTO* Obesity-Risk Variants

*Iroquois* homeobox (*Irx*) genes encode a family of highly conserved TALE homeodomain-containing transcription factors (TFs). *Iroquois* genes were first discovered in the fruit fly *Drosophila melanogaster.* Mutations of these genes suppress bristle formation on the lateral notum, leaving only a wide band of bristles in the central part of the notum, reminiscent of the hairstyle of the *Iroquois* American Indians “Mohawk” - hence the name of the locus (Gomez-Skarmeta and Modolell, 1996; Gomez-Skarmeta et al., 1996; Leyns et al., 1996). Mammals have 6 *Irx* genes clustered in two 3-gene groups; the IrxA cluster on mouse chromosome 13 (human chromosome 5) consists of *Irx1*, *Irx2*, and *Irx4*, and the IrxB cluster on mouse chromosome 8 (human chromosome 16) contains *Irx3*, *Irx5*, and *Irx6* (Peters et al., 2000; Cavodeassi et al., 2001; Kim et al., 2012). Among them, *Irx3* and *Irx5* of the IrxB cluster are involved in the development of many mammalian tissues including the heart, bone, limb bud, eye and ovary, with regulatory functions in embryonic patterning and specification as well as in tissue differentiation and specialization (Cheng et al., 2005; Costantini et al., 2005; Zhang et al., 2011; Gaborit et al., 2012; Kim et al., 2012; Li et al., 2014; Kim et al., 2016; Fu et al., 2018; Tan et al., 2020; Tao et al., 2020). *Irx3* and *Irx5* share similar expression patterns during embryonic development and their double mutant mice show more severe phenotypes than single mutants, suggesting that they possess overlapping function and act redundantly.

**FIGURE 1:**
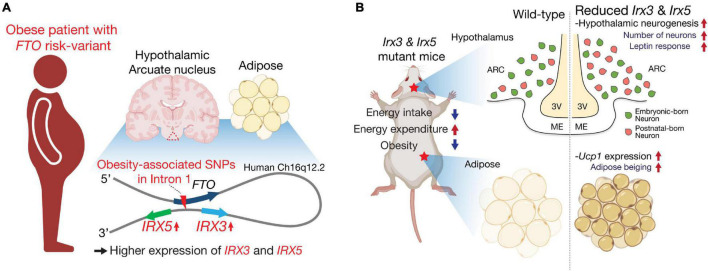
*IRX3* and *IRX5* are effectors of *FTO* obesity-risk variants and key determinants of energy homeostasis. **(A)** *IRX3* and *IRX5* are located about a half-megabase downstream of *FTO*. In human brain, particularly hypothalamic arcuate nucleus (triangle with dashed lines in red) and preadipocytes, they mediate the effects of obesity-associated variants located in the first intron of *FTO* through long-range interactions. **(B)** Mice with mutations in *Irx3* and/or *Irx5* exhibit an anti-obesity phenotype with increased energy expenditure and reduced food intake. These metabolic phenotypes are related to beiging of white adipose tissue and improved response to leptin as well as increased postnatal neurogenesis in the mediobasal hypothalamus. Schematics were created using illustrations from https://biorender.com.

Besides their developmental functions, *IRX3* and *IRX5* have been highlighted as determinants of human obesity in connection with the intronic obesity risk variants of *FTO*, a gene in the vicinity of the IRXB cluster. Non-coding variations represented by SNPs in the first intron of *FTO* are the strongest genetic risk factors of polygenic obesity in humans (Dina et al., 2007; Frayling et al., 2007; Cecil et al., 2008; Speakman et al., 2008; Tanofsky-Kraff et al., 2009). Previous studies using chromatin conformation capture techniques (3C, 4C and Hi-C) have demonstrated that a genomic region including the intronic *FTO* SNPs directly loops into the promoter region of the neighboring *IRX3* and *IRX5*, ∼ a half megabase downstream of *FTO*, in multiple tissues including adipose tissue and brain in humans (Figure 1A; Smemo et al., 2014; Claussnitzer et al., 2015; Sobreira et al., 2021). Furthermore, expression quantitative trait loci (eQTL) analysis revealed significantly higher expression levels of *IRX3* and *IRX5* in human brain, human hypothalamic arcuate-like neurons derived from human induced pluripotent stem cells (hiPSCs) and human adipocyte progenitor cells from risk-allele carriers, compared to non-risk allele carriers, whereas risk and non-risk carriers did not show significant difference of *FTO* expression (Figure 1A). Gene editing experiments in human adipocytes showed that an obesity-risk intronic variant of *FTO* results in higher expression of both *IRX3* and *IRX5* specifically in adipocyte progenitor cells (Claussnitzer et al., 2015), and deletion of a cis-regulatory module (CRM) harboring the *FTO* risk variant in mice results in lower *Irx3* and *Irx5* gene expression in adipocyte progenitors to roughly 70% of control levels (Laber et al., 2021). Furthermore, ∼20k-bp deletion spanning the orthologous obesity-associated interval of *Fto* in mice leads to down-regulation of both *Irx3* and *Irx5* in preadipocytes and developing hypothalamus (Sobreira et al., 2021). Collectively, these studies suggest that obesity-associated variants of *FTO* regulate the expression of *IRX3* and *IRX5* in the hypothalamus and adipose tissue contributing to metabolic changes.

## The Importance of *IRX3* and *IRX5* in Metabolic Regulation

Studies of genetic mouse models have demonstrated that *Irx3* and *Irx5* are both involved in metabolic regulation. Although there are differences in the metabolic phenotypes depending on the models, *Irx3-* and *Irx5*-deficient mice are generally lean and display an anti-obesity phenotype with elevated energy expenditure due to adipose beiging (i.e., enhanced adipose thermogenesis) compared to wild-type controls (summarized in Table 1). Specifically, *Irx3* knockout (*Irx3*^*tLz*/^*^*tLz*^*) and *Irx5* knockout (*Irx5*^*eGFP*/^*^*eGFP*^*) mice generated by our group both exhibited lower body weight and fat composition (Smemo et al., 2014; Bjune et al., 2018), whereas their heterozygous counterparts *Irx3*^*tLZ*/+^ and *Irx5*^*eGFP*/+^ mice did not exhibit any changes in body weight and composition (Son et al., 2021a). In a recent report, another line of *Irx3* knockout mice (*Irx3*^Δ/^*^Δ^*) generated by CRISPR editing was also shown to be lean and protected against diet-induced obesity (Sobreira et al., 2021). However, in the same study, mice homozygous for a new deletion mutation of *Irx5* (*Irx5*^Δ/^*^Δ^*) exhibited early postnatal lethality, whereas heterozygous *Irx5*^Δ/^*^+^* mutant mice were found to show an anti-obesity phenotype like *Irx3* knockout mice. While this discrepancy of *Irx5* mutant phenotypes remains to be investigated, genetic background and/or deletion of specific genomic sequences affecting the expression of neighboring genes could be contributing factors.

**TABLE 1:** Summary of metabolic studies with *Irx3* or *Irx5* mutant mice.

**Mouse model**	**Transgenic system**	**Target tissue**		**Metabolic phenotype**		**References**
*Irx3* ^*tLZ/tLZ*^	Part of exon 1 replaced by *tauLacZ*	Germ-line	Lean (Runty)	Energy expenditure ↑ Adipose beging ↑	*Food intake↓	Smemo et al., 2014
*Irx3* ^Δ/Δ^	CRISPR/Cas9 editing of exon 2	Germ-line	Lean	Adipose beging ↑	Sucrose preference↓	Sobreira et al., 2021
*Irx5* ^*eGFP/eGFP*^	Part of exon 1 replaced by *eGFP*	Germ-line	Lean (Runty)	Energy expenditure ↑ Adipose beging ↑	*Food intake↓	Bjune et al., 2018; Son et al., 2021b
*Irx3^+^Irx5^+^ /Irx3* ^*del*^ *Irx5* ^*eGFP*^	Deletion of exons 2 to 4 of *Irx3* in *Irx5*^*eGFP*^ line	Germ-line	Lean	Energy expenditure ↑ Adipose beging ↑	Food intake↓ Hypothalamic leptin response ↑	Son et al., 2021a
*Irx5* ^Δ/+^	CRISPR/Cas9 editing of exon 2	Germ-line	Lean	Adipose beging ↑	N/A	Sobreira et al., 2021
*Ins2-Cre;Rosa26^*EnR–Irx*3/+^*	Expression of dominant-negative form of IRX3	Hypothalamus	Lean	Energy expenditure ↑ Adipose beging ↑	*Food intake↓	Smemo et al., 2014; Son et al., 2021b
*Ins2-Cre;Irx3^*F/F*^*	Flanking exons 2 to 4 with *loxP* sites	Hypothalamus	Lean	No difference in energy expenditure	Food intake↓ Hypothalamic leptin response ↑	Son et al., 2021a

*Deletion of *Irx3* in macrophage, or expression of a “dominant-negative form of mouse *IRX3*” in adipose tissue (*aP2-Cre*;*Rosa26^*EnR–Irx3*^*^/+^) or “human *IRX3*” in brown adipose tissue (*Ucp1*-*Cre*;*Rosa26^*hIRX3*^*^/+^) leads to a lean phenotype with adipose beiging and elevated energy expenditure (Smemo et al., 2014; Yao et al., 2021; Zhang et al., 2021).*

*Though difference in daily food intake is not significantly different, these mutants show reduced amounts of food intake from long-term feeding analysis over life time.

Due to the requirement of *Irx3* and *Irx5* in the development of many organs, including heart (Costantini et al., 2005; Zhang et al., 2011; Gaborit et al., 2012; Kim et al., 2016) and bone (Cain et al., 2016; Bai et al., 2019; Tan et al., 2020) which are also important for energy homeostasis, whole body knockout mice of *Irx3* and *Irx5* are both runty and thus not ideal for studying metabolic regulation. To circumvent this, a mouse line harboring *cis*-heterozygous mutant allele of *Irx3* and *Irx5* (*Irx3*^+^*Irx5*^+^/*Irx3*^Δ^*Irx5*^*eGFP*^; *Irx3/5*^*dHet*^) was employed to study the effects of half-reduction of the expression of *Irx3* and *Irx5* on energy homeostasis (Son et al., 2021a). As obesity-risk variants of *FTO* affect the expression levels of both *IRX3* and *IRX5* (Claussnitzer et al., 2015; Laber et al., 2021; Sobreira et al., 2021), *Irx3/5*^*dHet*^ mice serve as a preclinical model mimicking lower *IRX3* and *IRX5* expression levels in humans. Unlike *Irx3*^*tLZ*/^*^*tLZ*^* and *Irx5*^*eGFP*/^*^*eGFP*^* mice, *Irx3/5*^*dHet*^ mice do not show a runty phenotype and develop with a normal body length at weaning (Son et al., 2021a). Nonetheless, *Irx3/5*^*dHet*^ mice display lower body weight and fat mass (Figure 1B), indicating that the dosage of *Irx3* and *Irx5* is critical for body mass regulation and suggesting that *Irx3* and *Irx5* play overlapping functions in energy homeostasis. Body weight and mass composition are determined by the balance of energy (food) intake and energy expenditure. *Irx3/5*^*dHet*^ mice show reduced food intake as well as elevated energy homeostasis (Table 1). Specifically, they exhibit beiging of white adipose tissue (WAT) with upregulation of brown adipocyte markers including *Ucp1* in WAT. In the hypothalamus, reduction of *Irx3* and *Irx5* dosage results in elevated neurogenesis during the early postnatal period leading to an increased number of orexigenic and anorexigenic arcuate neurons (Son et al., 2021a). Owing to the higher number of these leptin-sensing arcuate neurons, *Irx3/5*^*dHet*^ mice display enhanced hypothalamic leptin response and reduced food intake. Indeed, reduced food intake is a key feature of the metabolic phenotypes of *Irx3/5*^*dHet*^ mice as these mutant mice still exhibit a body weight significantly lower than their control counterparts at thermoneutrality, when the effects of adipose thermogenesis and difference in energy expenditure are minimized (Son et al., 2021a). These observations suggest that higher expression of *IRX3* and *IRX5* could lead to elevated food intake contributing to human obesity, which is mostly associated with excessive energy consumption.

The hypothalamic function of *Irx3* in energy homeostasis has been examined in transgenic as well as conditional knockout models using *Ins2-Cre*, which targets specific hypothalamic cells with high *Irx3* and *Irx5* expression (see below). *Ins2-Cre*;*Rosa26*^*EnR–Irx3*^ mice with hypothalamic expression of a dominant-negative form of *Irx3* (EnR-Irx3; full length IRX3 protein fused to the Engrailed transcriptional repressor domain) exhibit a strong metabolic phenotype with reduced food intake and elevated energy expenditure, similar to those of *Irx3*^*tLz*/^*^*tLz*^* and *Irx5*^*eGFP*/^*^*eGFP*^* mice (Table 1; Smemo et al., 2014). In contrast, *Ins2-Cre*;*Irx3*^*Flox/Flox*^ mice with specific hypothalamic deletion of *Irx3* display a less pronounced metabolic phenotype; they are not as lean as *Ins2-Cre*;*Rosa26*^*EnR–Irx3*^ mice and do not exhibit any adipose beiging/energy expenditure phenotypes (Table 1). Specifically, the phenotypes of *Ins2-Cre*;*Irx3*^*Flox/Flox*^ mice include reduced food intake, enhanced hypothalamic leptin response, and elevated neurogenesis in the postnatal hypothalamus, which are also observed in *Irx3/5*^*dHet*^ mice (Figure 1B; Son et al., 2021a). Together, these results suggest a hypothalamic function of *Irx3* as well as *Irx5* in feeding regulation through the control of postnatal neurogenesis. As most clinical data have illustrated that the risk alleles of *FTO* are associated with increased energy intake, our data highlight hypothalamic postnatal neurogenesis regulated by *IRX3* and *IRX5* as a potential mechanism affecting leptin response in human obesity.

## Expression of *IRX3* and *IRX5* in Radial Glia-Like Cells of the Postnatal Mouse Hypothalamus

The hypothalamic ARC-ME contains diverse cell types involved in energy homeostasis (Timper and Bruning, 2017). de Araujo et al. (2019) reported that *Irx3* is expressed in a subset of POMC neurons from the analysis of a public single-cell RNA-sequencing (scRNA-seq) dataset of ∼20,000 hypothalamic ARC-ME cells and suggested that POMC neuronal expression of *Irx3* is important for the regulation of energy homeostasis (Campbell et al., 2017; de Araujo et al., 2019). On the contrary, neuronal expression of *Irx3* and *Irx5* is minimal in the postnatal mouse hypothalamus. Neither *Irx3* nor *Irx5* expression is detectable in the published transcriptional profiles of POMC neurons and AgRP neurons (Henry et al., 2015). Furthermore, in a recently published scRNA-seq dataset by Romanov et al., which profiled gene expression of the whole hypothalamus at diverse developmental timepoints from embryonic day (E) 15.5 to postnatal day (P) 23, *Irx3* and *Irx5* are almost exclusively expressed in radial glial cells at postnatal stages though their expression can be found in neurons of the posterior hypothalamus at embryonic timepoints (Romanov et al., 2020).

**FIGURE 2:**
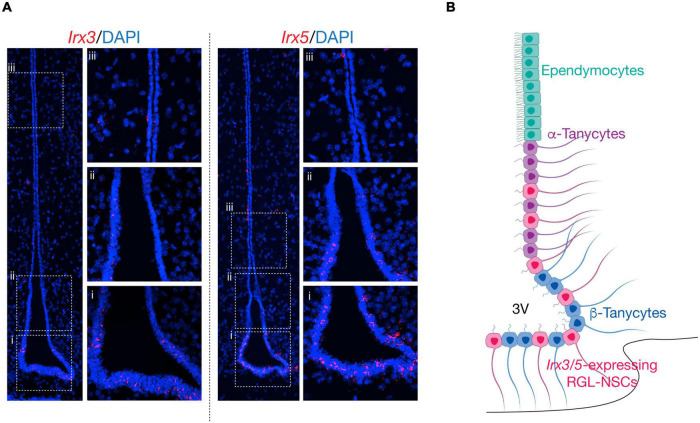
Schematic of *Irx3* and *Irx5* expression in the mediobasal hypothalamus. **(A)** RNA *in situ* hybridization of *Irx3* and *Irx5* the mediobasal hypothalamus at postnatal day (P) 14. *Irx3* and *Irx5* expression is mainly found in radial glia-like cells lining the third ventricle, including RGL-NSCs, tanycytes, and ependymocytes. Panels from bottom to top (ventral to dorsal) show representative regions from: β-tanycytes (i), α-tanycytes (ii) and ependymocytes (iii). **(B)** Schematic of the mediobasal hypothalamus. Along the third ventricle, ependymocytes are located at the dorsal ventricular walls, followed by α-tanycytes, and β-tanycytes. *Irx3*- and *Irx5*-expressing RGL-NSCs are interspersed among α-tanycytes and β-tanycytes in the dorsal ventricular wall and floor.

In our recent study, analysis of publicly available scRNA-seq datasets from adult hypothalamic ARC-ME revealed the expression of *Irx3* and *Irx5* mostly in RGLs including ependymocytes and tanycytes, as well as in some NG2^+^ OPCs. By RNA *in situ* hybridization, *Irx3* and *Irx5* expression are found along the wall of the third ventricle which harbors both ependymocytes and tanycytes (Figures 2A,B). Furthermore, by scRNA-seq analysis of *Ins2-Cre*;*tdTomato*^+^ cells from P10 ARC-ME, which include cells lining the third ventricle and their descendants in the ARC-ME, the expression of *Irx3* and *Irx5* is mainly detected in a novel RGL-NSC population in addition to tanycytes, ependymocytes, and NG2^+^ OPCs. Our data illustrate that *Irx3* and *Irx5* are predominantly expressed in the RGL-NSCs, which are distinct from tanycytes, and behave as neural progenitors in the postnatal mouse hypothalamus. Importantly, the function of *Irx3* in postnatal neurogenesis in the hypothalamic ARC-ME and feeding regulation has been established by conditional deletion in these cells and their descendants in *Ins2-Cre*;*Irx3*^*Flox/Flox*^ mice (Son et al., 2021a). Before discussing the potential functions of *Irx3* and *Irx5* in RGL-NSCs, we provide below a brief review of postnatal-adult neurogenesis and description of different RGL cell types residing in the hypothalamus.

## Postnatal-Adult Neurogenesis in the Hypothalamus

Neurogenesis is a complex and highly regulated process that results in the generation of new neurons. It occurs at high rates during the embryonic period when substantial quantities of new cells are generated by the proliferation/differentiation of neural precursor cells (NPCs, also known as radial glia cells (RGCs) as these NPCs possess astrocyte-like radial processes) (Gotz and Huttner, 2005; Taverna et al., 2014; Obernier and Alvarez-Buylla, 2019). Over the past decades, studies in multiple mammalian species have illustrated that substantial levels of neurogenesis persist in specific adult brain areas, known as neurogenic niches (Dimou and Gotz, 2014). Postnatal-adult neurogenesis corresponds to the series of events that lead to the production of new neurons in the postnatal-adult brain, from precursor cell division to the survival and functional integration of newly differentiated neurons (Lledo et al., 2006). Adult neurogenesis in the mouse brain starts around the second postnatal week (P14), when radial glia cells shift sharply from an embryonic to an adult state with distinct molecular identity from that of their embryonic progenitors, and continues throughout life (Hochgerner et al., 2018; Ghosh, 2019). In the adult mouse brain under normal conditions, well-characterized niches are mainly located in the subventricular zone (SVZ) of lateral ventricles (Doetsch et al., 1999; Magavi et al., 2000) and the hippocampal subgranular zone (SGZ) (Altman and Das, 1965). However, there are evidence suggesting constitutive neurogenesis also in the proximity of circumventricular organs including hypothalamus (Kokoeva et al., 2005, 2007; Hourai and Miyata, 2013).

**FIGURE 3:**
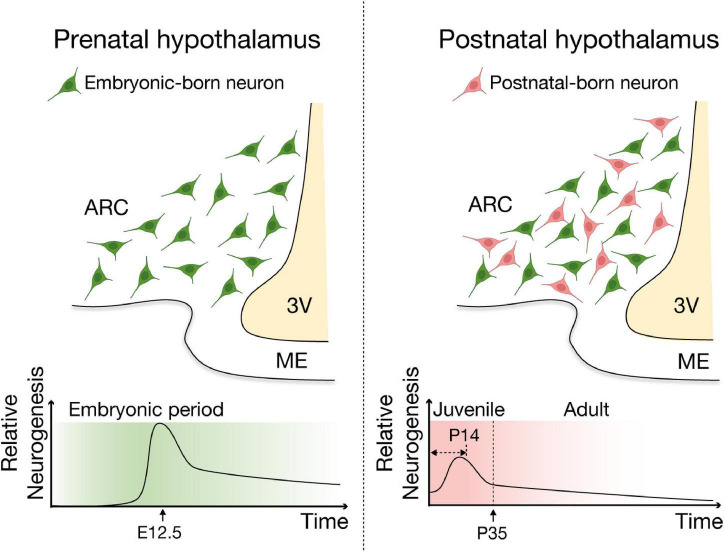
Schematic of embryonic and postnatal neurogenesis in the ARC-ME of the hypothalamus. Two major waves of neurogenesis have been reported in the hypothalamus. In mice, the neurogenic period of the developing hypothalamus encompasses embryonic day (E) 10 to E16, with a peak of neurogenesis at around E12.5. A second wave of neurogenesis is detected during the juvenile stages, especially within the first two postnatal weeks and continues throughout adulthood and contributes remodeling of the hypothalamic neuronal circuit. This figure was adapted from Son et al. (2021a) with modification.

Development of hypothalamic neuronal system starts during the embryonic period (Padilla et al., 2010; Ishii and Bouret, 2012; MacKay and Abizaid, 2014). In mice, hypothalamic neurons are mainly born during the embryonic period with a sharp peak of neurogenesis occurring around E12.5 (Padilla et al., 2010; Ishii and Bouret, 2012), followed by postnatal neurogenesis that actively contributes to the remodeling of hypothalamic neuronal population in juvenile (young adult; between P15 and P35) and adult mice (Lee et al., 2012; McNay et al., 2012; Haan et al., 2013; Yoo and Blackshaw, 2018; Figure 3). Although the basal (unstimulated) levels of neurogenesis in the adult hypothalamus are probably low and less than those seen in the well-established neurogenic niches, SVZ and SGZ (Lledo et al., 2006; Migaud et al., 2010), neurogenesis in the hypothalamus continues throughout adulthood and contributes to remodeling of the hypothalamic neuronal circuit. Early *in vitro* studies reported that cells dissociated from rodent hypothalamus, including the parenchymal regions and the ependymal layer of the third ventricle, proliferate, form neurospheres, and subsequently differentiate into both neural and glial lineages (Evans et al., 2002; Markakis et al., 2004; Xu et al., 2005), supporting the notion that third ventricle wall and its vicinity, particularly hypothalamic ARC-ME, are neurogenic niches in the adult mouse brain. These findings have been corroborated by studies using 5-bromo-2′-deoxyuridine (BrdU) or 5′-iodo-2′-deoxyuridine (IdU) labeling of proliferating cells as well as cell lineage analysis (Gotz and Huttner, 2005; Kokoeva et al., 2007; Lee et al., 2012; Li et al., 2012; McNay et al., 2012; Haan et al., 2013; Robins et al., 2013). ARC neurons exhibited substantial turnover in the adult mouse, with more than half of those neurons found at 4 weeks of age being replaced over the following 8 weeks (Pierce and Xu, 2010; Lee et al., 2012; Li et al., 2012; McNay et al., 2012). These adult-born hypothalamic neurons functionally integrate into the neuronal circuitry (Robins et al., 2013) and physiological responses including neuroendocrine regulation (e.g., release neurotransmitter/neuropeptides and leptin sensing) (Kokoeva et al., 2005; Pierce and Xu, 2010; Haan et al., 2013; Son et al., 2021a), suggesting adult neurogenesis likely contributes to other hypothalamic functions such as regulation of energy homeostasis.

## Radial Glia-Like Cells in Postnatal Hypothalamic Neurogenesis

In the SVZ and SGZ, postnatal and adult NPCs are also known as RGLs, which share the characteristics of embryonic RGCs, including radial morphology, apical contact with the brain ventricles and expression of stemness markers (Urban and Guillemot, 2014; Morales and Mira, 2019). They are capable of undergoing both self-renewal through cell division and generating other neural cell types through differentiation. Besides these two neurogenic niches, RGLs also exist in other CNS regions, such as in the retina, where they are known as Muller glia (Goldman, 2014), and in some circumventricular organs, such as the median eminence of the hypothalamus (Bennett et al., 2009; Hourai and Miyata, 2013; Furube et al., 2020).

### Tanycytes, Neural Progenitors in Postnatal Hypothalamic Neurogenesis

Tanycytes are specialized RGLs with a single elongated basal process that projects toward the hypothalamus parenchyma. They reside in the hypothalamus ventricular layer but are distinct from the cuboid and multi-ciliated ependymocytes. Tanycytes originate from the infundibulum of the mesencephalon, which is a local extension of the neuroepithelium located directly above the Rathke’s pouch (or future pituitary) (Pearson and Placzek, 2013; Rizzoti and Lovell-Badge, 2017). The LIM homeodomain transcription factor *Lhx2* is critical for tanycyte specification and development, through activation and maintenance of the tanycyte-specific transcription factor *Rax*, and inhibition of the ependymocyte gene *Rarres2* (Miranda-Angulo et al., 2014; Salvatierra et al., 2014). In rats, tanycytes arise perinatally and terminally differentiate during the first postnatal month (Rodriguez et al., 2005). In mice, the expression of tanycyte markers *Rax* and *Gpr50* are first detected by RNA *in situ* hybridization around E14.5 in the ventricular zone of the mesencephalon, likely corresponding to “pre-tanycytes” (Shimogori et al., 2010). Recent scRNA-seq studies have identified a cell type closely resembling hypothalamic RGCs around E10 that has the potential to differentiate into multiple neural progenitors (Kim et al., 2020; Romanov et al., 2020; Zhang et al., 2021). Though tanycytes and ependymocytes are likely derived from these hypothalamic RGCs at late embryonic stages, their lineages appear to diverge as early as E13, as demonstrated by the expression of their respective cell type specific markers. Candidate factors, such as *Wnt7b*, *Hopx*, *Ptch1*, *Nfib* and *Foxj1*, are suggested to be implicated in this process (Kim et al., 2020). Along the same lines, Zhang et al. (2021) proposed a “state-switching” model, where a subpopulation of hypothalamic RGCs switch into tanycyte precursors at an early developmental stage (∼E11) and suggested that a substantial fraction of “pre-tanycytes” are generated in parallel to prenatal neurogenesis (Zhang et al., 2021).

Based on their dorsal-ventral location along the ventricular layer, marker gene expression and functions, tanycytes can be divided into four major subtypes, namely α1, α2, β1, and β2 (reviewed in Rodriguez et al., 2005; Goodman and Hajihosseini, 2015; Rizzoti and Lovell-Badge, 2017; Yoo and Blackshaw, 2018). Tanycytes not only play important regulatory roles in energy balance (Yoo et al., 2020), transport of nutrients and hormones between the blood-hypothalamus barrier (Mullier et al., 2010; Langlet et al., 2013; Balland et al., 2014; Miranda-Angulo et al., 2014; Duquenne et al., 2021), but also express canonical markers of adult NSCs in the SVZ and SGZ, including *Nestin*, *Sox2*, *Vimentin*, etc. (Rodriguez et al., 2005; Ming and Song, 2011; Lee et al., 2012; Bolborea and Dale, 2013; Goodman and Hajihosseini, 2015; Campbell et al., 2017; Yoo and Blackshaw, 2018).

Genetic lineage tracing has served as a powerful tool in uncovering the functional heterogeneity of neural precursor cells in postnatal and adult mouse hypothalamus as well as mapping their fate and differentiation potential. Table 2 summarizes various *Cre* mouse lines that have been used to target different hypothalamic neural precursors, where typically *Cre* recombinase is driven by a gene promoter of interest at a specific timepoint, allowing for the induction and expression of a fluorescent protein (or other markers) in the targeted neural stem/progenitor cells and its descendant progeny in the hypothalamus. In combination with BrdU labeling, these lineage tracing experiments highlight the proliferative ability of ventricular zone in the postnatal mouse hypothalamus. By using *Rax-CreER* (targeting all tanycytes), tanycytes were shown to give rise to both neurons and glia in the postnatal and juvenile mouse hypothalamus (Pak et al., 2014; Yoo et al., 2021). These findings were further supported by scRNA-seq analysis of *Rax-CreER* lineage cells (Yoo et al., 2021). However, it is worth noting that the neurogenic and gliogenic abilities of *Rax*^+^ tanycytes appear to be drastically reduced at adult stage, as tanycytes labeled by *Rax-CreER* at P60 generate very few neurons under physiological conditions (Pak et al., 2014).

**TABLE 2:** Summary of lineage tracing studies of postnatal hypothalamic neurogenesis from radial glia-like cells.

**Mouse model**	**Main target cell type**	**Study design**	**Neurogenic potential**	**Gliogenic potential**	**References**
*Nestin-CreER; Rosa26* ^*YFP*^	β2-Tanycytes	Induction method: 4-Hydroxytamoxifen Timepoint: P4 or P7 Chase: 30d	Yes ME neurons (Hu^+^)	Not assesed	Lee et al., 2012
*Nestin-CreERT2; CAG* ^*tdTomato*^	Ependymal cells	Induction method: Tamoxifen Timepoint: P90 Chase: short-term 30d; long-term 180d, 390d	Yes Peak at 9-16 month old	Yes Short-term: ependymal cells Long-term: astrocytes (GFAP^+^)	Chaker et al., 2016
*Fgf10-CreERT2;Rosa26^*LacZ*^*	β-Tanycytes	Induction method: Tamoxifen Timepoint: post-wean P28-32, adult P53-P83 Chase: short-term 10d; long-term 22-72d	Yes Long-term: neurons in ARC, VMH, LH	Yes Short-term: mostly tanycytes Long-term: ependymal cells	Haan et al., 2013
*Fgf10-CreERT2;Rosa26^*tdTomato*^*	β-Tanycytes	Induction method: Tamoxifen Timepoint: P28 Chase: short-term 3d; long-term 8-27d	Yes Long-term: neurons in ARC, VMH, LH	Yes	Haan et al., 2013
*Fgf10-CreERT2;Rosa26^*tdTomato*^*	β-Tanycytes	Induction method: Tamoxifen Timepoint: P5 Chase: short-term 3d, 5d, 7d, 14d; long-term 65-85d	Yes Short-term: neurons in ARC, VMH, DMH after 7-14d chase Long-term: retention of parenchymal neurons	Yes Short-term: ependymal cells Long-term:parenchymal astrocytes and oligodendrocytes	Goodman et al., 2020
*Prss56-Cre;Rosa26^*tdTomato*^* (or *Rosa26^*mTmG*^)*	α2-Tanycytes	Cre onset timepoint: P14 Chase: 75d	Yes Mainly neurons in ARC and DMH	Yes α2-Tanycytes and parenchymal astrocytes in ARC and DMH	Jourdon et al., 2016
*GLAST-CreERT2; Rosa26* ^*Z/EG*^	α-Tanycytes	Induction method: Tamoxifen Timepoint: P42-P56 Chase: short-term 5d, 10d; long-term 21d, 42d, 252d	Yes Long-term: neurons in ARC and VMH	Yes Short-term: mostly α2-tanycytes Long-term: α-tanycytes, β-tanycytes and parenchymal astrocytes in ARC, VMH	Robins et al., 2013
*Rax-CreERT2; CAG^*tdTomato*^*	Tanycytes	Induction method: 4-Hydroxytamoxifen Timepoint: P7, P28, P50 Chase: 3d, 5d, 10d	Potentially immature parenchymal neurons	Yes Tanycytes (Vim^+^, Sox2^+^) and potentially parenchymal glia	Pak et al., 2014
*Rax-CreERT2; Rosa26* ^*tdTomato*^	Tanycytes	Induction method: Tamoxifen Timepoint: P60 Chase: 3d, 7d, 14d, 30d	Not under physiological conditions	Not under physiological conditions	Mu et al., 2021
*Rax-CreER; CAG^*Sun*1–GFP^*	Tanycytes	Induction method: 4-Hydroxytamoxifen Timepoint: P3-P5 Chase: short-term 14d; long-term 40d	Yes Short-term: ME neurons Long-term: parenchymal neurons in ARC, VMH, DMH	Yes Short-term: tanycytes Long-term: tanycytes and parenchymal astrocytes, OPCs in ARC, VMH, DMH	Yoo et al., 2021
*Sox2-GFP/Cre; Rosa26* ^*YFP*^	Hypothalamic NSCs	Induction method: Lentivirus injection to mediobasal hypothalamus Timepoint: P90 Chase: short-term 5d; long-term 80d	Yes Long-term: ARC neurons (including NPY and POMC neurons)	Yes Long-term: astrocytes (S100B^+^) and oligodendrocytes (RIP^+^) in ARC	Li et al., 2012
*Ins2-Cre; Rosa26* ^*tdTomato*^	RGL-NSCs	Cre onset timepoint: P5 Chase: 5d, 16d, 40d	Yes Mainly neurons in ARC	Yes Ependymal cells along ventricle and parenchymal OPCs/oligodendrocytes in ARC	Son et al., 2021a

These lineage tracing studies support the idea of RGL heterogeneity in the postnatal mouse hypothalamus and indicate that different RGL subtypes have distinct proliferation/differentiation abilities. Specifically, α-tanycytes, which are located at the lateral walls of the third ventricle, can be labeled by *GLAST-Cre* and appear to be mostly gliogenic with restricted neurogenic potential (Haan et al., 2013; Robins et al., 2013). They have limited self-renewal properties, generating new α- and β-tanycytes in the hypothalamic ventricular region (Robins et al., 2013). On the other hand, ventrally located β-tanycytes, such as *Fgf10*-labeled β-tanycytes and *Nestin*-labeled β2-tanycytes, show higher neurogenic potentials adding new neurons mainly to the VMH/ARC and ME, respectively (Lee et al., 2012; Haan et al., 2013; Goodman et al., 2020). Recently, *Fgf10*-labeled β-tanycytes have also been demonstrated to have the ability to give rise to a proliferating population of α-tanycytes that later undergoes both symmetric and asymmetric divisions to generate parenchymal daughter cells, suggesting that α-tanycytes can act as transient amplifying/intermediate cells during postnatal hypothalamic neurogenesis (Goodman et al., 2020).

### Novel Radial Glia-Like Neural Stem Cells in the Postnatal ARC

Through lineage tracing and scRNA-seq analyses using *Ins2-Cre*, Son et al. (2021a) unveiled a previously unreported RGL cell type, termed as radial glia-like NSCs (RGL-NSCs), which line the hypothalamic third ventricle from around P5 (Son et al., 2021a). At P10, there is a rapid expansion of *Ins2-Cre* labeled cells in the parenchyma of the ARC, which gradually plateau after P21. Importantly, among the *Ins2-Cre* labeled cells in the parenchyma of adult ARC, over 85% of them are Hu^+^ neurons, and about one third of leptin-responsive AgRP/NPY and POMC/CART neurons in the adult ARC are derived from the *Ins2-Cre* lineage. These results suggest that the novel RGL-NSCs possess robust neurogenic capability in early postnatal mouse hypothalamus and corroborate with those of previous studies (Pierce and Xu, 2010; McNay et al., 2012) illustrating the presence of rapid and continuous neuronal turnover in the postnatal mouse hypothalamus.

**FIGURE 4:**
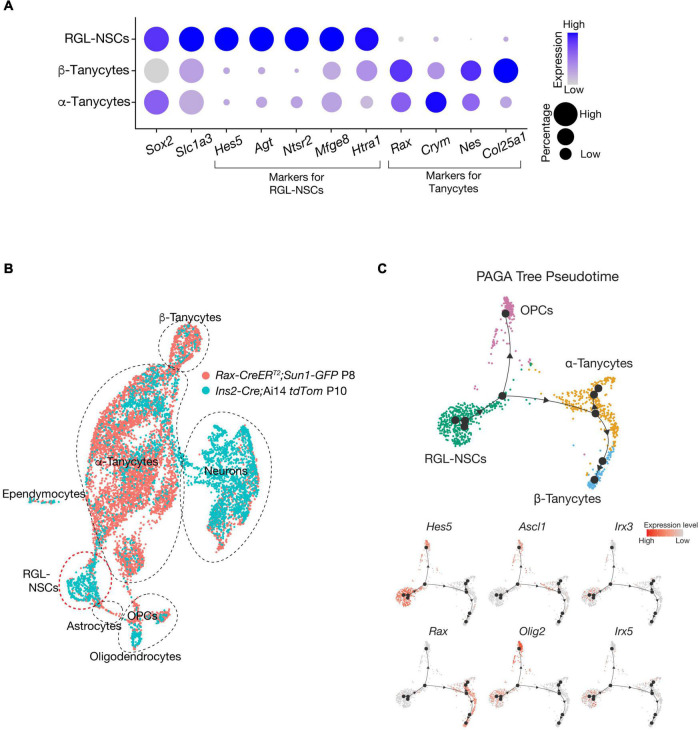
*Irx3* and *Irx5* expressing RGL-NSCs are distinct from tanycytes. **(A)** Expression of cell type-specific markers in RGL-NSCs, α-tanycytes, and β-tanycytes from the *Ins2-Cre*;Ai14 *tdTomato* scRNA-seq dataset. The size of the dot indicates the percentage of cells expressing a specific gene in each cluster, and the color represents the average expression level of the gene. RGL-NSCs and tanycytes share expression of the broad stemness marker *Sox2* and glial marker *Slc1a3* (also known as GLAST), while NSCs markers such as *Hes5*, *Agt*, *Ntsr2* are almost exclusively found in RGL-NSCs, and canonical tanycytes markers such as *Rax* and *Crym* are absent in RGL-NSCs. **(B)** Integrated UMAP plot of *Rax-CreER*^*T2*^;*Sun1-GFP* at P8 (*Cre* recombinase activity induced between P3 and P5) (GSE160378) and *Ins2-Cre*;Ai14 *tdTomato* at P10 (GSE154969) scRNA-seq datasets through the reference-based integration feature of Seurat and UMAP visualization. The RGL-NSC cluster consists of cells originating from the *Ins2-Cre*;Ai14 *tdTomato* dataset, and does not overlap with tanycytes nor astrocytes. Also note that while a much larger amount of tanycytes are detected in the *Rax-CreER*^*T2*^;*Sun1-GFP* dataset, *Ins2-Cre*;Ai14 *tdTomato* is more robust at labeling neurons. **(C)** Pseudo-temporal analysis of *Ins2-Cre* labeled RGL-NSCs, tanycytes and OPCs using Partition-based graph abstraction (PAGA) Tree. The line with arrowhead indicates the inferred differentiation trajectory, which originates from *Hes5*-expressing RGL-NSCs, undergoes a bifurcated differentiation path to *Olig2*-expressing OPCs and *Rax*-expressing tanycytes. The NSC activation marker *Ascl1* is detected in bridge cells but not in RGL-NSCs, suggesting their quiescence.

Noticeably, when *Cre* activities are induced at a similar postnatal timepoint (∼P3-P5), while *Rax-CreERT* and other *Cre* mouse lines, such *as Fgf10-Cre*, efficiently label tanycytes and their descendant progenies in the VMH and DMH, *Ins2-Cre* appears to be more ideal for marking newly generated neurons residing in the ARC and ME regions of postnatal mouse hypothalamus. In addition, these RGL-NSCs are molecularly distinct from tanycytes of the mouse hypothalamus, as they do not express the canonical markers, such as *Rax* and *Crym* (Figure 4A). Indeed, comparison with *Rax-CreERT* marked cells (Yoo et al., 2021) at similar postnatal timepoints using a ‘reference-based integration’ analysis with the single-cell R toolkit Seurat (Stuart et al., 2019) reveals minimal overlap between the progeny of *Rax-CreERT* labeled tanycytes and those of RGL-NSCs (Figure 4B). By pseudo-temporal analysis (Wolf et al., 2019), RGL-NSCs are inferred to possess the potential of differentiating into both tanycyte and OPC lineages (Figure 4C). Interestingly, the expression of *Irx3* and *Irx5* appears to be downregulated upon differentiation of RGL-NSCs (Figure 4C). These RGL-NSCs display strong expression of stemness as well as astroglia markers including *Sox2*, *Hes5*, *Agt*, etc., which are features reminiscent of the adult NSCs in the SVZ and SGZ (Figure 4A). In particular, their transcriptome resembles those of the *Sox2-eGFP* NSCs from the SVZ of adult mouse brain (Shah et al., 2018). Furthermore, these hypothalamic RGL-NSCs also show high expression of neurogenic TFs including *Pax6, Nkx2.2*, and *Olig2*, which are consistent with their purported roles in neuron and oligodendrocyte differentiation.

By profiling the transcriptome of the whole hypothalamus at multiple developmental timepoints from E15.5 to P23 using scRNA-seq, Romanov et al. (2020) have determined a comprehensive single cell map of the developing hypothalamus and identified a population of RGCs marked by *Sox2* and *Hes5* expression at both E15.5 and E17.5. These RGCs appear to be drastically diminished after birth and are apparently replaced by a postnatal type of RGL with elevated expression of several adult NSC markers including *Agt*, *Htra1*, *Ntsr2* and *Mfge8* (Yuzwa et al., 2017; Hochgerner et al., 2018; Shah et al., 2018; Zywitza et al., 2018). Intriguingly, the transcriptional profiles of the RGL-NSCs labeled by *Ins2-Cre* are highly similar to those of the postnatal RGLs identified by Romanov et al. (2020), but distinct from those of the embryonic RGCs. Together, these observations suggest that RGL-NSCs represent a postnatal type of RGL with robust neurogenic potential and *Ins2-Cre* can be used for enriching these postnatal hypothalamic NSCs in the mouse brain.

## Regulatory Factors of Hypothalamic Postnatal Neurogenesis

Factors, such as hormonal signals, environment, and genetics, could affect postnatal neurogenesis in the hypothalamus. Specifically, remodeling of ARC through elevated neurogenesis by treatment with ciliary neurotrophic factor (CNTF) could lead to leptin-dependent weight loss and reduced food intake (Kokoeva et al., 2005). Secreted factors including epidermal growth factor (EGF), basic fibroblast growth factor (bFGF), insulin-like growth factor I (IGF-I) and brain-derived neurotrophic factor (BDNF) also could significantly stimulate proliferation, survival and development of newborn neurons in the adult stage (Pencea et al., 2001; Xu et al., 2005; Perez-Martin et al., 2010; Robins et al., 2013; Chaker et al., 2016).

Given the importance of the hypothalamus in controlling energy homeostasis, several studies using rodent models have evaluated the environmental effects on neurogenesis. In this aspect, perhaps the most widely studied factor is high fat diet (HFD), which has been reproducibly shown to impair survival of hypothalamic NSCs and inhibit neurogenesis in the hypothalamus (Lee et al., 2012, 2014; Li et al., 2012; McNay et al., 2012). For example, Li et al. (2012) reported that the neurogenic impairment of *Sox2*^+^ hypothalamic NSCs from chronic HFD treatment is associated with overactivation of the pro-inflammatory pathway involving IκB kinase β (IKKβ) and its downstream nuclear factor κB (NF-κB), as well as paracrine release of cytokines from neighboring microglia. By *in vitro* studies, they further suggested that IKKβ/NF-κB inhibit neuronal differentiation of these hypothalamic NSCs through direct activation of downstream Notch signaling (Li et al., 2012). Intriguingly, neurogenesis in the ME is increased in response to HFD in female mice illustrating that there is an apparent sex-difference in the control of hypothalamic neurogenesis (Lee et al., 2012, 2014). Although scRNA-seq analysis suggest that young male and female adult mice have a similar proportion of tanycytes and other neural cell types under normal diet (Campbell et al., 2017), their distinct responses to HFD could be attributed to multiple factors, such as sex hormone levels, pubertal neurogenesis in sexually dimorphic brain regions, penetrance of the blood-brain barrier, etc. (Ahmed et al., 2008; Lee et al., 2014). Furthermore, caloric restriction is associated with a tendency of reduced neurogenesis within the ME (Lee et al., 2014). On the other hand, heat exposure and physical activity facilitate proliferation of neuronal progenitor cells in the hypothalamus and promotes differentiation to neurons (Matsuzaki et al., 2009; Niwa et al., 2016).

In addition to extrinsic factors, genetic studies have begun to unveil the role of intrinsic factors in the control of postnatal ARC neurogenesis. For example, deletion of the nuclear factor I family of transcription factors (*Nfia*/*b*/*x*) in *Rax*^+^ tanycytes results in activation of Shh and Wnt signaling, which in turn stimulates proliferation of tanycytes and their robust neurogenic differentiation in postnatal mouse hypothalamus (Yoo et al., 2021). This study highlights the inhibitory functions of *Nfia*/*b*/*x* TFs in restricting the neurogenic differentiation of tanycytes and suggests the involvement of Shh and Wnt signaling in the control of hypothalamic postnatal neurogenesis. Goodman et al. (2020) reported that *Fgf10* acts as a negative regulator of postnatal neurogenesis in mouse hypothalamus (Goodman et al., 2020). Conditional loss of *Fgf10* in β-tanycytes retards their amplification, which is compensated by retention and delay of their transient intermediate progeny, eventually leading to increased number of parenchymal neurons in the VMH, DMH and ARC. Recently, Son et al. (2021a) demonstrated the regulatory functions of *Irx3* and *Irx5* in hypothalamic postnatal neurogenesis and ARC remodeling affecting leptin response. Specifically, *Irx3/5*^*dHet*^ mice as well as *Ins2-Cre;Irx3*^*Flox/Flox*^ mice with specific deletion of *Irx3* in the RGL-NSCs both exhibit elevated neurogenesis in the postnatal ARC, leading to higher number of leptin-sensing neurons (Son et al., 2021a).

## Activation of Neural Stem Cells in Postnatal Hypothalamus

**FIGURE 5:**
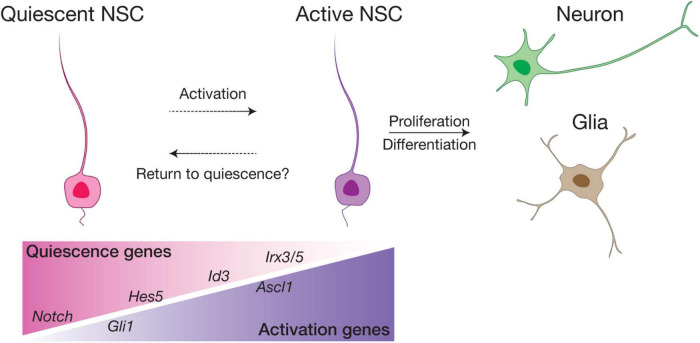
Schematic model of NSC quiescence and activation. In the SVZ and SGZ neurogenic niches, the majority of adult NSCs are quiescent and can be activated by various factors to proliferate and differentiate into neurons and glia. We postulate that in the ventral hypothalamus, RGL-NSCs are mostly in a quiescence state enriched in *Notch* ligand, *Hes5* and *Id3* expression. *Irx3* and *Irx5* are likely novel factors involved in maintaining the quiescence of RGL-NSCs in the postnatal hypothalamus. Sub-populations of primed RGL-NSCs expressing *Gli1*, and active NSCs marked by *Ascl1* also exist in small proportions.

In the SVZ and SGZ of adult mouse brain, the majority of RGLs are mitotically inactive and remain largely in G0 (out of the cell cycle), a fundamental characteristic that distinguishes them from embryonic RGCs (Morales and Mira, 2019). These quiescent NSCs (qNSCs) can be activated (active NSCs, or aNSCs) to re-enter the cell cycle, becoming either rapidly proliferating intermediate progenitor cells (IPC) that subsequently give rise to newborn neuronal cells, or returning to quiescence (Figure 5). Quiescence of NSCs is typically marked by astroglia features (*Agt*, *ApoE*, *Aldh1h*, etc.) with high *Hes* and *Id* gene expression, while factors associated with aNSCs include *Fgfr3*, *Ascl1* and cell cycle genes (e.g., *cyclinD*) (Shin et al., 2015; Urban et al., 2016; Morales and Mira, 2019; Obernier and Alvarez-Buylla, 2019; Urban et al., 2019). Using a combination of fate mapping and scRNA-seq analyses, recent studies have further suggested the presence of additional states between quiescence and active, such as “dormant” (or deeply quiescent) and “primed” states for qNSCs, as well as early, mid or late stages for aNSCs, illustrating the functional and molecular heterogeneity of adult NSCs (Dulken et al., 2017; Basak et al., 2018; Hochgerner et al., 2018; Shah et al., 2018; Bottes et al., 2021).

The balance between quiescence and activity is crucial for the long-term maintenance of adult NSC pools and prevention of tumors resulting from over-proliferation. Thus, this process is tightly regulated by multiple factors. Perhaps the most extensively studied signaling pathway in this context is Notch signaling, which plays a pivotal role in adult neurogenesis, promoting both quiescence and proliferation of NSCs. Deletion of the Notch effector gene *Rbpj* as well as inhibition of Notch signaling activate qNSCs, resulting in an immediate burst of neurogenesis, but ultimately lead to exhaustion of the NSC pool (Ehm et al., 2010; Imayoshi et al., 2010; Engler et al., 2018). On the other hand, activation of Notch signaling induces the expression of *Hes* and *Hey* families of TFs, which act together with Id factors, to maintain quiescence of NSCs through repression of the proneural TF *Ascl1* (Bai et al., 2007; Urban et al., 2016; Boareto et al., 2017; Harris and Guillemot, 2019; Sueda et al., 2019). Shh signaling is another developmental pathway that has been implicated in postnatal and adult NSCs of the SVZ (Ahn and Joyner, 2005; Faigle and Song, 2013; Petrova et al., 2013; Daynac et al., 2016), where various components of the Shh signaling pathway are expressed. The use of *Gli1-CreER* mice illustrated that qNSCs and aNSCs of both SVZ and SGZ are Shh-responsive (Ahn and Joyner, 2005). Furthermore, a recent study using a combination of genetic fate mapping and transcriptome profiling studies revealed that *Gli1*^+^ qNSCs represent a long-term self-renewing qNSC subpopulation in the SGZ contributing to neurogenesis of the hippocampus (Bottes et al., 2021).

**FIGURE 6:**
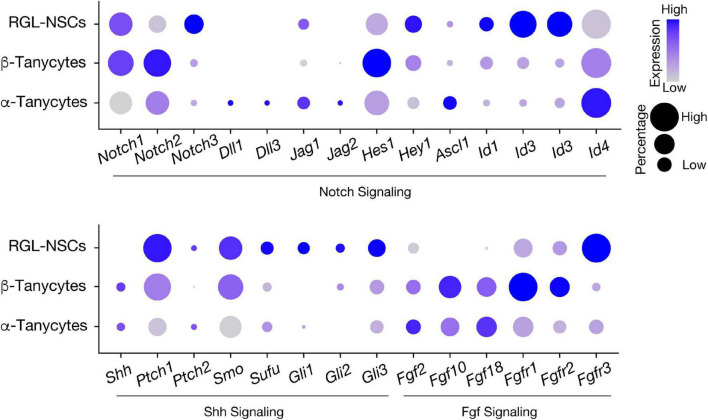
Expression of signaling pathway genes involved in neural stem cell activation in hypothalamic RGLs. Expression of Notch, Shh, and Fgf pathway-related genes in RGL-NSCs, α-tanycytes, and β-tanycytes from the *Ins2-Cre*;Ai14 *tdTomato* scRNA-seq dataset. The size of the dot indicates the percentage of cells expressing a specific gene in each cluster, and the color represents the average expression level of the gene.

In our recent study, bioinformatics analysis has suggested that RGL-NSCs shift from quiescence to a more active state upon reduction of *Irx3* and *Irx5* dosage, which is consistent with the observation of elevated neurogenesis in *Irx3/5*^*dHet*^ mice (Son et al., 2021a). These findings imply that *Irx3* and *Irx5* negatively regulate NSC activation and postnatal neurogenesis. Based on their transcriptomic profiles, most *Ins2-Cre*-labeled RGL-NSCs are quiescent with high expression of *Hes* and *Id* genes, and only very few cells show *Ascl1* expression, representing a small population of aNSCs (Figure 6). Intriguingly, many Shh pathway genes are expressed in a subset of the RGL-NSCs indicating that *Ins2-Cre*-labeled RGL-NSCs might represent another Shh-responsive cell population in the postnatal brain; the membrane receptors (*Ptch1* and *Smo*) as well as the intracellular mediators of Shh signaling (*Sufu*, *Gli1*, *Gli2* and *Gli3*) are expressed in the RGL-NSC population, whereas the *Shh* ligand is expressed in neighboring tanycytes (Figure 6). Relevant to this, *Irx3* and several neural progenitor genes, including *Pax6*, *Nkx2.2* and *Olig2*, are known to be regulated by Shh signaling during ventral neural tube development (Briscoe et al., 2000; Qi et al., 2001; Zhou et al., 2001), and all these neural progenitor genes are also expressed in the RGL-NSCs. Furthermore, multiple studies have suggested that *Irx* genes are involved in early development of the central nervous system (Bellefroid et al., 1998; Gomez-Skarmeta et al., 1998; Cavodeassi et al., 2001). In the developing embryonic mouse brain, *Irx3* and *Irx5* might regulate the expression of its putative target *Ascl1* (Cohen et al., 2000), which is a key regulator of embryonic neurogenesis in the hypothalamus (McNay et al., 2006; Alvarez-Bolado, 2019). Our recent study also suggests that *Irx3* and *Irx5* could affect postnatal hypothalamic neurogenesis through cell cycle regulation of RGL-NSCs (Son et al., 2021a). Consistent with this notion, reduction of *Iro* proteins promotes cell proliferation by accelerating G1 to S transition in *Drosophila* imaginal disks (Barrios et al., 2015) and loss of both *Irx3* and *Irx5* results in a higher proportion of G2/M cells with a reduction of G1 cells in the developing mouse limb bud (Tao et al., 2020).

## Concluding Remarks and Future Directions

*IRX3* and *IRX5* have emerged as a strong link between the non-coding genetic variations of the *FTO* gene and obesity. Using genetic mouse models, work by us and others depicts their critical roles in metabolic regulation in the brain, as well as in peripheral organs such as adipose tissue (Smemo et al., 2014; Claussnitzer et al., 2015; Bjune et al., 2018; Sobreira et al., 2021; Son et al., 2021a,b). More recently, macrophage *Irx3* has also been associated with metabolic inflammation and body weight control (Yao et al., 2021). In this review, we summarize the metabolic functions and regulatory mechanisms of *Irx3* and *Irx5* with a focus on the postnatal hypothalamus, where they are highly expressed, particularly in the newly identified RGL-NSCs giving rise to postnatal hypothalamic neurons, and regulate energy homeostasis. Recent technical advances such as chronic *in vivo* labeling, visualization tool, genetic lineage tracing, single-cell RNA sequencing and bioinformatic analysis have greatly contributed to our improved understanding of the hypothalamic neural precursor cells, and their contribution to hypothalamic neurogenesis. Using these approaches, we demonstrate that *Ins2-Cre*-labeled RGL-NSCs represent a *bona fide* population of NSCs in the postnatal mouse hypothalamus. Through differentiation into tanycytes and NG2^+^ OPCs, this novel NSC population likely contributes to both neuron and oligodendrocyte lineages in the hypothalamic ARC-ME, respectively.

Nonetheless, there are many remaining questions about RGL-NSCs and additional characterization is required. What factors regulate their stem cell behaviors and what are the molecular actions of *Irx3* and *Irx5* in these cells? Do they originate from a similar population of embryonic RGCs as tanycytes and ependymocytes, and what are the genes involved in their fate specification and divergence from other hypothalamic RGLs? Are they still present and functional in the adult and aging mouse hypothalamus?

While our current knowledge of hypothalamic RGL-NSCs is still limited, especially as their lineage relationship with other hypothalamic cell types is mainly based on computational analyses and lineage tracing experiments using the *Ins2-Cre* mouse line, additional *in vivo* fate mapping studies as well as clonal analysis using inducible labeling techniques specific to the RGL-NSC population are warranted. For example, the *Hes5-CreERT2* mouse line, which has been previously used in mapping the NSC lineage of the hippocampal SGZ (Lugert et al., 2012), could be a powerful genetic tool to further investigate the lineage relationships between RGL-NSCs and other hypothalamic cell types such as tanycytes and NG2^+^ OPCs. In the hypothalamus, *Hes5* is a marker of RGL-NSCs but is not found in other RGLs such as tanycytes, allowing for specific labeling of the RGL-NSC progeny (Figure 4A; Son et al., 2021a). In addition, the inducible nature of this mouse line, which is not available with the *Ins2-Cre* mouse line, will also enable targeting of RGL-NSCs at various timepoints during hypothalamus development, and investigating the functions of *Irx3* and *Irx5* as well as other regulatory factors in these RGL-NSCs in a spatiotemporal manner. At the same time, the lessons learnt from the neurogenic niches in SVZ and SGZ should provide us with many useful hints about potential genetic and environmental factors involved in the regulation of this novel hypothalamic NSC population. As RGL-NSCs and tanycytes display high expression of Notch, Shh and Fgf pathway genes (Figure 6), future studies focusing on these signaling pathways in the regulation of RGL-NSC behavior and hypothalamic neurogenesis in juvenile and adult mice will be informative. Furthermore, by defining the genes/pathways regulated by *Irx3* and *Irx5* in RGL-NSCs as well as their interplays with the Notch, Shh and Fgf pathways should unveil new knowledge about postnatal remodeling of the hypothalamus and provide mechanistic insights in NSC biology and obesity.

## Author Contributions

ZD, JS, and C-CH conceived, designed, and wrote the manuscript. All authors contributed to the article and approved the submitted version.

## Conflict of Interest

The authors declare that the research was conducted in the absence of any commercial or financial relationships that could be construed as a potential conflict of interest.

## Publisher’s Note

All claims expressed in this article are solely those of the authors and do not necessarily represent those of their affiliated organizations, or those of the publisher, the editors and the reviewers. Any product that may be evaluated in this article, or claim that may be made by its manufacturer, is not guaranteed or endorsed by the publisher.
